# Antiangiogenic Agents in Combination with Chemotherapy in Patients with Advanced Non-Small Cell Lung Cancer

**DOI:** 10.3109/07357907.2011.554476

**Published:** 2011-04-06

**Authors:** Susanna V Ulahannan, Julie R Brahmer

**Affiliations:** Department of Oncology, Sidney Kimmel Comprehensive Cancer Center, Johns Hopkins University Hospital, Baltimore, Maryland, USA

**Keywords:** Antiangiogenesis inhibitors, Non-small cell lung cancer, Bevacizumab

## Abstract

Most patients with non-small cell lung cancer (NSCLC) present with advanced disease requiring systemic chemotherapy. Treatment with the antiangiogenic agent bevacizumab in combination with standard platinum-based doublet chemotherapy has been shown to improve outcomes in patients with advanced NSCLC. Several multitargeted antiangiogenic tyrosine kinase inhibitors (e.g., sorafenib, sunitinib, cediranib, vandetanib, BIBF 1120, pazopanib, and axitinib) are also being evaluated in combination with standard chemotherapy. Here we review current clinical data with combination therapy involving antiangiogenic agents and cytotoxic chemotherapy in patients with advanced NSCLC.

## INTRODUCTION

Lung cancer remains the leading cause of cancer-related deaths in the United States, with approximately 219,440 new cases and 159,390 deaths expected in 2009 (1). Each year more deaths result from lung cancer than from breast, col-orectal, and prostate cancers combined (2). Approximately 85% of all lung cancer cases are categorized as non-small cell lung cancer (NSCLC), and most patients present with advanced disease at the time of diagnosis (1, 3). The standard of care for patients with advanced disease is platinum-based doublet chemotherapy (4). Adding a third cytotoxic agent to the regimen increases toxicity and does not provide additional clinical benefits (4). The Eastern Cooperative Oncology Group (ECOG) conducted a large (N = 1,207) randomized study that compared four platinum-based doublet chemotherapy regimens in patients with NSCLC (5). None of the regimens was found to yield superior efficacy, though fewer episodes of toxicity were noted with the combination of carboplatin and paclitaxel (5). The median survival in this study was 8 months (5). Although there is a survival benefit with improved quality of life when chemotherapy is given to patients with advanced NSCLC, it appears that an efficacy plateau is reached when conventional chemotherapy is used alone.

Angiogenesis is the growth of new microvessels from preexisting vasculature, a process that involves a fine balance of proangiogenic and antiangiogenic factors and coordination between multiple cell types such as macrophages, en-dothelial cells, and pericytes (6-9). Angiogenesis is necessary for cancer cells to proliferate beyond microscopic size and to metastasize (10). The vasculature associated with pathologic angiogenesis is abnormal in structure and function; it is characterized by tortuous, dilated, saccular vessels that are poorly organized and hyperpermeable (6-8). These vascular abnormalities lead to an abnormal tumor microenvironment with interstitial hypertension, hypoxia, and acidosis; this, in turn, increases the production of vascular endothelial growth factor (VEGF) and decreases the effectiveness of cytotoxic chemotherapy (11, 12).

The vascular endothelial growth factor plays a key role in regulation, both in normal and cancer cells, promoting endothelial cell migration and proliferation necessary for angiogenesis. VEGF is over expressed in a majority of malignant tumors, including NSCLC (12-15), and elevated blood levels of VEGF are associated with tumor aggressiveness and a poor prognosis (13). Three VEGF receptors (VEGFR) have been identified: VEGFR1, VEGFR2, and VEGFR3. The biologic effects of VEGF are mediated by VEGFR1 and VEGFR2; VEGFR2 is believed to play the primary role in activating endothelial cells. VEGFR3 is associated primarily with lymphatic vessel growth (12, 14). Other growth factors, such as platelet-derived growth factor (PDGF) and fibroblast growth factor (FGF), also play key roles in promoting angiogenesis (16, 17).

Bevacizumab is a monoclonal antibody that targets circulating VEGF and inhibits VEGF binding to VEGFRs, thereby preventing its proangiogenic activity (18). In 2006, bevacizumab was approved by the US Food and Drug Administration for the first-line treatment of patients with advanced nonsquamous NSCLC in combination with carboplatin and paclitaxel (19). However, resistance often develops, and only approximately 50% of patients are actually eligible for bevacizumab treatment. Several mechanisms have been proposed that may account for the additive or synergistic activity of antiangiogenic agents and cytotoxic chemotherapy, including the possibility that anti-VEGF therapy may transiently normalize leaky tumor vascula-ture,which could facilitate more effective drug delivery to the tumor (15). Although bevacizumab is currently the only approved antiangiogenic agent for patients with NSCLC, other agents are in clinical development. These agents have been evaluated in combination with a variety of chemotherapeutic drugs for the treatment of patients with NSCLC. In this review, we focus on the use of combination therapy with antiangiogenic agents and chemotherapy in patients with advanced NSCLC.

## TARGETING ANGIOGENESIS WITH BEVACIZUMAB

### First-line treatment

Bevacizumab is the first antiangiogenesis agent to show a survival benefit when added to standard doublet chemotherapy in the first-line treatment of patients with advanced NSCLC (20). A randomized phase II trial of 99 patients with advanced NSCLC compared paclitaxel and carboplatin therapy with or without bevacizumab 7.5 or 15 mg/kg (Table 1) (21). The patients who received the higher dose of bevacizumab had a higher response rate (RR) (31.5% vs. 18.8%), longer time to progression (TTP; 7.4 months vs. 4.2 months; *p* = .023), and a trend toward increased overall survival (OS) (17.7 months vs. 14.9 months;p = .63) compared with patients given placebo. However, fatal hemoptysis was observed in four of 66 bevacizumab-treated patients and was apparently associated with squamous cell histology, tumor cavita-tion, centrally located tumors, and tumors close to major vessels (21).

**Table 1:** Results from phase II and III trials of bevacizumab in combination with chemotherapy as first-line or second-line NSCLC treatment

Reference	Phase	Regimen	N^a^	RR (%)	PFS (mo)	OS (mo)
*First-line*						
Johnson 2004 [21]	II	CBDCA AUC 6 + PTX 200 mg/m^2^	32	18.8	*TTP*, 4.2	14.9
		CBDCA + PTX + Bev 7.5 mg/kg	32	28.1	*TTP*, 4.3	11.6
		CBDCA + PTX + Bev 15 mg/kg	35	31.5	*TTP*, 7.4	17.7
Patel 2009 [22]	II	CBDCA AUC 6 + Pem 500 mg/m^2^ + Bev 15 mg/kg with maintenance Pem 500 mg/m^2^ + Bev 15 mg/kg	49	55	7.8	14.1
Skaff 2009 [23]	II	CBDCA AUC 6 + Pem 500 mg/m^2^ + Bev 15 mg/kg	25	36	*TTP*, 7.25	ND
Dalsania 2007 [24]	II	CBDCA AUC 6 + Pem 500 mg/m^2^ + Bev 15 mg/kg	12	60	ND	ND
Waples 2008 [25]	II	OX 120 mg/m^2^ + Pem 500 mg/m^2^ + Bev 15 mg/kg	58	26	7.8	16.7
Wozniak 2009 [26]	II	Pem 500 mg/m^2^ + Gem 1500 mg/m^2^ + Bev 10 mg/kg every 2 weeks	18	61	ND	ND
Lilenbaum 2008 [27]	II	OX 130 mg/m^2^ + Gem 1,000 mg/m^2^ + Bev 15 mg/kg	44	43	ND	13.7
Leon 2009 [28]	II	CDDP 80 mg/m^2^ + Vinorelbine 25 mg/m^2^ + Bev 15 mg/kg	17	29.4	4.6	ND
Herbst 2007 [29]	II	TXT 75 mg/m^2^ or Pem 500 mg/m^2^ + Bev 15 mg/kg	40	12.5	4.8	12.6
		TXT 75 mg/m^2^ or Pem 500 mg/m^2^ + placebo	41	12.2	3.0	8.6
		Bev 15 mg/kg + Erlotinib 150 mg/day	39	17.9	4.4	13.7
Ferrer 2009 [30]	II	CDDP 75 mg/m^2^ + TXT 75 mg/m^2^ + Bev 15 mg/kg	46	63	7.8	13.5
William 2010 [30]	II	CBDCA AUC 6 + TXT 75 mg/m^2^ + Bev 15 mg/kg	40	52.5	7.9	16.5
Reynolds 2009 [31]	II	nabPTX 300 mg/m^2^ CBDCA AUC 6 + Bev 15 mg/kg	50	31	9.8	16.8
Clement-Duchene 2010 [32]	II	CBDCA AUC 5 + Gem 1000 mg/m^2^ + Bev 15 mg/kg	47	14.9	8.7	12.8
Sandler 2006 [20]	III	CBDCA AUC 6 + PTX 200 mg/m^2^	433	15	4.5	10.3
		CBDCA + PTX + Bev 15 mg/kg	417	35	6.2	12.3
Reck 2009 [33]	III	CDDP 80 mg/m^2^ + Gem 1,250 mg/m^2^	327	20.1	6.1	NR
		CDDP + Gem + Bev 7.5 mg/kg	330	34.1	6.7	NR
		CDDP + Gem + Bev 15 mg/kg	329	30.4	6.5	NR
Crino 2010 [34]	III	Chemotherapy + Bev 7.5 or 15 mg/kg	2, 212	51.0	*TTP*, 7.8	14.6
Fischbach 2009 [35]	III	Chemotherapy + Bev 7.5 or 15 mg/kg	1,758	ND	6.7	ND
*Second-line*						
Adjei 2010 [36]	II	Pem 500 mg/m^2^ + Bev 15 mg/kg	48	10	4.0	8.6
Heist 2008 [37]	II	Pem 500 mg/m^2^ + OX 120 mg/m^2^ + Bev 15 mg/kg	34	27	5.8	12.5

a Patients evaluable for efficacy.

*Abbreviations*. AUC: area under the curve; Bev: bevacizumab; CBDCA: carboplatin; CDDP: cisplatin, EFS: event-free survival;Gem: gemcitabine;ND: no data available; NSCLC: non-small cell lung cancer;OS: overall survival; OX: oxaliplatin; Pem: pemetrexed; PFS: progression-free survival; PTX: paclitaxel; RR: response rate; TTP: time to progression; TXT: docetaxel.

Subsequently, ECOG conducted a large, randomized, multicenter, phase III study (E4599) that enrolled 878 patients with advanced or recurrent nonsquamous NSCLC (Table 1) (20). Carboplatin/paclitaxel was administered every 3 weeks for six cycles with or without bevacizumab 15 mg/kg (20). Treatment with bevacizumab was continued until evidence of disease progression. In order to reduce the risk of bleeding, patients with squamous cell histology, brain metastases, therapeutic anticoagulation, or a history of gross hemoptysis were excluded from the trial. The primary end point, OS, was statistically superior in patients who received bevacizumab (12.3 months vs. 10.3 months; hazard ratio [HR], 0.79; *p* = .003) (20). These patients also showed a significant improvement in RR (35% vs. 15%; *p* < .001) and progression-free survival (PFS) (6.2 months vs. 4.5 months; *p* < .001) (20). Increased frequencies of bleeding, febrile neutropenia, hypertension, and proteinuria were reported in the bevacizumab arm (*p* < .05). There was also a higher incidence of treatment-related deaths in patients given bevacizumab than in patients given chemotherapy alone (15 vs. *2;p* = .001) (20). The 15 deaths in the bevacizumab arm were attributed to pulmonary hemorrhage (N = 5), complications of neutropenic fever (N = 5), gastrointestinal (GI) bleeding (N = 2), cerebrovascular events (N = 2), and a probable pulmonary embolus (N = 1) (20). Bevacizumab was subsequently approved based on the results of this trial.

The retrospective analyses from E4599 revealed that OS was not significantly improved with bevacizumab in women (20). However, OS with or without bevacizumab was higher in women than in men, though this difference did not reach statistical significance (20). There was no difference in OS in patients of >70 years of age, but they did have a higher degree of reported toxicity (38). The biomarkers VEGF, basic FGF, intercellular adhesion molecule (ICAM), and E-selectin were measured before and after treatment in E4599 (39). Low baseline ICAM levels were significantly associated with improved RR (32% vs. 14% in patients with high ICAM levels; *p* = .02) and OS *(p* = .00005) (39). This suggests that patients with low baseline ICAM levels could benefit from the addition of bevacizumab to standard chemotherapy regimens; however, this needs to be confirmed in prospective randomized trials.

A second phase III, randomized trial, AVAiL, evaluated bevacizumab 7.5 mg/kg and 15 mg/kg in combination with cisplatin and gemcitabine in patients with advanced nonsquamous NSCLC (Table 1) (33). This study showed significant improvement in the primary end point, PFS, with the addition of bevacizumab at either the high dose (6.5 months vs. 6.1 months; HR, 0.82; *p =* .03) or the low dose (6.7 months vs. 6.1 months; HR, 0.75; *p =* .003) compared with chemotherapy alone, at a median follow-up of >7 months (33). Response rates in the patients receiving high-dose bevacizumab, low-dose bevacizumab, and placebo were 30.4% *(p* = .0023), 34.1% *(p* < .0001), and 20.1%, respectively (33). After a median of >12.5 months of follow-up, median OS was not significantly different from chemotherapy alone with bevacizumab 7.5 mg/kg (13.1 months vs. 13.6 months; HR, *0.93;p* = .42) or 15 mg/kg (13.1 months vs. 13.4 months; HR, 1.03; *p* = .761) (40). Although AVAiL trial was not powered to directly compare the two doses of bevacizumab, the results indicate similar efficacy and toxicity profiles (33). A retrospective analysis found that either dose of bevacizumab used as single-agent maintenance therapy might have clinical benefit (PFS, 4.6 months vs. 3.2 months with control), although bevacizumab was not associated with an OS benefit (41).

Activity was also observed in a phase II study with the combination of pemetrexed, carboplatin, and bevacizumab followed by maintenance therapy with pemetrexed and bevacizumab as first-line treatment in patients with advanced NSCLC (Table 1) (22). In the 49 patients assessed, RR was 55%, PFS was 7.8 months, and OS was 14.1 months. No grade 3/4 hypertension or pulmonary hemorrhage was observed, but four cases of grade 3/4 diverticulitis were reported (22). This was a small trial that included more women than men, which could explain the favorable survival rate. In light of the data from this trial, the large (N = 900) phase III Pointbreak trial was initiated to compare (a) pemetrexed, carboplatin, and bevacizumab followed by maintenance therapy with pemetrexed and bevacizumab with (b) paclitaxel, carboplatin, and bevacizumab followed by maintenance bevacizumab (42). Several other phase II trials are evaluating the combination of bevacizumab with platinum-based doublet chemotherapy as first-line treatment in patients with advanced NSCLC (Table 1) (22, 23-28, 30-32, 43). In addition, many other clinical studies are currently recruiting patients and will evaluate first-line bevacizumab in combination with chemotherapy and/or pemetrexed (Table 2).

**Table 2:** Overview of ongoing (actively recruiting) phase II and III trials of bevacizumab in NSCLC^a^

Phase	Trial description	Identifier number
*Early-stage NSCLC*		
II	Neoadjuvant bevacizumab in combination with cisplatin-based chemotherapy versus neoadjuvant cisplatin and docetaxel, both followed by adjuvant bevacizumab, in patients with Stage IB-IIIA NSCLC undergoing surgical resection.	NCT00130780
II	Neoadjuvant bevacizumab in combination with carboplatin and paclitaxel in patients with Stage IB-IIA NSCLC undergoing surgical resection.	NCT00960297
II	Neoadjuvant bevacizumab in combination with cisplatin and gemcitabine, followed by cisplatin and etoposide after surgical resection, in patients with Stage IIIA NSCLC.	NCT00924209
II	Adjuvant bevacizumab in combination with carboplatin and docetaxel, followed by maintenance bevacizumab and erlotinib, in patients with surgically resected Stage IB-IIIA NSCLC.	NCT00621049
III	Adjuvant bevacizumab in combination with chemotherapy versus chemotherapy alone in patients with surgically resected Stage IB-IIIA NSCLC.	NCT00324805
*First-line in advanced NSCLC*		
II	Bevacizumab in combination with carboplatin and gemcitabine.	NCT00150657
II	Bevacizumab in combination with carboplatin and gemcitabine.	NCT00400803
II	Bevacizumab in combination with carboplatin and abraxane.	NCT00642759
II	Bevacizumab in combination with carboplatin, paclitaxel, and erlotinib.	NCT00550537
II	Bevacizumab in combination with carboplatin, paclitaxel, and imprime PGG® injection.	NCT00874107
II	Bevacizumab in combination with paclitaxel and gemcitabine.	NCT00655850
II	Bevacizumab in combination with docetaxel in patients aged >75 years.	NCT00541099
II	Bevacizumab in combination with gemcitabine and cisplatin versus bevacizumab in combination with gemcitabine in patients aged .70 years.	NCT01077713
II	Bevacizumab in combination with platinum-based chemotherapy versus bevacizumab in combination with erlotinib.	NCT00531960
II	Bevacizumab in combination with carboplatin and paclitaxel versus bevacizumab in combination with carboplatin, paclitaxel, and erlotinib in nonsmokers.	NCT00976677
II	Bevacizumab in combination with carboplatin and paclitaxel versus bevacizumab in combination with carboplatin, paclitaxel, and cixutumumab.	NCT00955305
II	Bevacizumab in combination with carboplatin and paclitaxel, versus CT-322 in combination with carboplatin and paclitaxel.	NCT00850577
II	Bevacizumab in combination with carboplatin and paclitaxel, or second-line bevacizumab in combination with erlotinib, in patients with asymptomatic untreated brain metastases.	NCT00800202
II	Bevacizumab in combination with pemetrexed.	NCT00254319
II	Bevacizumab in combination with paclitaxel and pemetrexed.	NCT00807573
II	Bevacizumab in combination with carboplatin and pemetrexed.	NCT00614822
II	Bevacizumab in combination with carboplatin and pemetrexed in patients with ECOG performance status 2.	NCT00892710
II	Bevacizumab in combination with cisplatin and pemetrexed.	NCT00998166
II	Bevacizumab in combination with gemcitabine and pemetrexed.	NCT00438204
II	Bevacizumab in combination with erlotinib in patients aged ≥70 years.	NCT00553800
II	Bevacizumab in combination with cisplatin and pemetrexed versus bevacizumab in combination with erlotinib.	NCT01116219
II	Bevacizumab in combination with cisplatin and pemetrexed, followed by maintenance bevacizumab and pemetrexed.	NCT01004250
II	Bevacizumab in combination with carboplatin and gemcitabine, followed by bevacizumab in combination with erlotinib at disease progression.	NCT00702975
II	Bevacizumab in combination with carboplatin and docetaxel, followed by second-line bevacizumab and pemetrexed or second-line pemetrexed alone.	NCT00766246
III	Carboplatin and paclitaxel with or without bevacizumab versus carboplatin, paclitaxel, and cetuximab with or without bevacizumab.	NCT00946712
III	Bevacizumab in combination with pemetrexed, versus bevacizumab alone and pemetrexed alone.	NCT01107626
III	Bevacizumab in combination with pemetrexed and carboplatin versus bevacizumab in combination with pemetrexed in patients aged >65 years.	NCT00976456
III	Bevacizumab in combination with cisplatin and pemetrexed, followed by maintenance bevacizumab and pemetrexed, versus maintenance bevacizumab alone.	NCT00961415
III	Bevacizumab in combination with carboplatin and pemetrexed, followed by maintenance bevacizumab and pemetrexed, versus first-line bevacizumab in combination with carboplatin and paclitaxel, followed by maintenance bevacizumab.	NCT00762034
III	Bevacizumab in combination with carboplatin and paclitaxel, followed by maintenance bevacizumab, versus first-line pemetrexed in combination with carboplatin, followed by maintenance pemetrexed.	NCT00948675
*Second- or third-line in advanced NSCLC*		
II	Second- or third-line bevacizumab in combination with carboplatin and paclitaxel.	NCT00753909
II	Second-line bevacizumab in combination with vinorelbine.	NCT00755170
II	Second-line bevacizumab in combination with docetaxel.	NCT00741195
II	Second-line bevacizumab in combination with pemetrexed.	NCT00741221
II	Second-line bevacizumab in combination with pemetrexed versus pemetrexed alone.	NCT00735891
II	Second-line bevacizumab in combination with erlotinib.	NCT00749567
II	Second-line bevacizumab in combination with erlotinib.	NCT00436332
II	Second-line bevacizumab in combination with ixabepilone.	NCT01057212

a ClinicalTrials.gov accessed on November 22, 2010.

Abbreviations. ECOG: Eastern Cooperative Oncology Group; NSCLC: non-small cell lung cancer.

### Second-line Treatment

Bevacizumab has also been studied as second-line therapy (Table 1). One phase II trial evaluated the efficacy and toxi-city of pemetrexed plus bevacizumab as second-line therapy in 48 patients with advanced NSCLC (36). A partial response (PR) was reported in five patients (10%) and stable disease (SD) was reported in 19 patients (40%), with a median PFS of 4.0 months and OS of 8.6 months (36). The grade 3/4 hematologic toxicities occurring in >10% of patients were neutropenia (19%), leukopenia (17%), and lymphopenia (13%) (36). The grade 3/4 nonhematologic toxicities occurring in >10% of patients were thrombosis (10%), dyspnea (10%), and fatigue (13%) (36). A separate phase II trial compared bevacizumab plus chemotherapy (docetaxel or pemetrexed), bevacizumab plus erlotinib, and chemotherapy alone in 120 patients with advanced nonsquamous NSCLC in the second-line treatment setting (29). Median PFS for bevacizumab-chemotherapy, bevacizumab-erlotinib, and chemotherapy alone was 4.8 months, 4.4 months, and 3.0 months, respectively, while median OS was 12.6 months, 13.7 months, and 8.6 months, respectively. There were no significant differences for these outcomes between the two bevacizumab arms, but superiority for disease progression or death was demonstrated for bevacizumab-chemotherapy versus chemotherapy alone (HR, 0.66, 95% CI, 0.38-1.16) and for bevacizumab-erlotinib versus chemotherapy alone (HR, 0.72, 95% CI, 0.42-1.23). Partial response or complete response (CR) was reported in five patients in each of the bevacizumab-chemotherapy and chemotherapy alone arms, and for seven patients in the bevacizumab-erlotinib arm (29). The grade 3/4 neutropenia occurred in eight patients receiving bevacizumab-chemotherapy, two patients receiving bevacizumab-erlotinib, and seven patients receiving chemotherapy alone.

These results and those of an earlier randomized phase III trial comparing pemetrexed and docetaxel suggest that the combination of bevacizumab and pemetrexed may provide clinical benefit in the treatment of NSCLC (44). Results from another phase II study suggest that the addition of oxaliplatin to bevacizumab/pemetrexed may further improve outcomes (Table 1) (37).

### Safety

The toxicities associated with bevacizumab may be directly related to its mechanism of action. Hypertension, which occurs frequently, may be due to the decreased synthesis of nitrous oxide that occurs as a result of VEGF inhibition and leads to increased vascular tone (45). In addition, hypertension induced by bevacizumab may also contribute to proteinuria (46).

Bevacizumab has been associated with a large number of potentially serious adverse events (AEs) in patients with NSCLC. The most serious, and sometimes fatal, are GI perforation, wound healing complications, hemorrhage, arterial thromboembolic events, hypertension, nephrotic syndrome, neutropenia, and congestive heart failure (47). Common AEs in patients receiving bevacizumab include asthenia, abdominal pain, other pain, headache, hypertension, diarrhea, nausea, vomiting, anorexia, stomatitis, constipation, upper respiratory infection, epistaxis, dyspnea, exfoliative dermatitis, and proteinuria (47).

The ATLAS trial of maintenance bevacizumab and er-lotinib (N = 598) (48), the PASSPORT trial of bevacizumab with first- or second-line chemotherapy (N = 106) (49), and the BeTa trial of bevacizumab with erlotinib in the second-line setting (N = 37) (50) all included patients with treated brain metastases, with some receiving therapeutic an-ticoagulation. Central nervous system (CNS) hemorrhages were reported in three patients participating in ATLAS, and five patients in ATLAS and three patients in PASSPORT-experienced pulmonary hemorrhages (48,49). These data indicate that patients with treated brain metastases and patients receiving therapeutic anticoagulation maybe treated with bevacizumab.

The most common AEs in AVAiL were hematologic and related to GI, with a similar incidence in the three treatment arms (low-dose bevacizumab, high-dose bevacizumab, and placebo) (51). Adverse events that occurred at a higher frequency with bevacizumab included hypertension (7% and 9% vs. 2%), proteinuria (2% and 3% vs. 0%), and bleeding (4% and 5% vs. 2%). Hemoptysis was reported in 0.5% and 1.2% of patients in the low- and high-dose bevacizumab arms and in 1.3% of patients in the placebo arm (51). Serious AEs were reported in 39%, 45%, and 36% of patients, respectively (51). Despite the fact that 9% of the study population was receiving therapeutic anticoagulation, no pulmonary hemorrhage was reported in the initial publication of the trial or the final safety analysis (33, 51).

Two large cohort studies (SAiL and ARIES) have focused on the safety of bevacizumab. SAiL, which enrolled 2,212 patients, evaluated the safety of first-line bevacizumab, 7.5 mg/kg and 15 mg/kg, in combination with chemotherapy. At baseline, 4% of the patients received anticoagulation therapy, with bleeding seen in 924 patients (34). However, significant bleeding and hemoptysis were rare and serious bleeding (grade >3) of any cause was reported in 81 patients. Arterial and venous thromboembolism occurred in 302 patients, and cerebral hemorrhage in seven patients (34). Congestive heart failure was observed in 17 patients. Hypertension occurred in 790 patients, but only 125 patients had grade >3 hypertension. Proteinuria was reported in 764 patients, and GI perforation was reported in 30 patients (34).

The ARIES trial (N = 1,518), which is evaluating bevacizumab in combination with first-line chemotherapy regimens, has enrolled patients with locally advanced or metastatic NSCLC. The most common first-line chemotherapeutic regimen used with bevacizumab was carboplatin/paclitaxel (64%) (35). Of the treated patients, 8% had brain metastases and 5% were receiving therapeutic anticoagulation. A total of 45 patients had a grade >3 bleeding event, one had CNS hemorrhage, and 22 had serious arterial thromboembolic events. Adverse effects in the overall population included hypertension (3.8%) and grade >3 bleeding events (GI hemorrhage, 1.1%; severe pulmonary hemorrhage, 0.7%; and CNS hemorrhage, 0.1%) (35).

Results from E4599 have suggested a longer OS in patients with hypertension (15.9 months vs. 11.5 months without hypertension) and improved PFS with the onset of hypertension during bevacizumab treatment (7.0 months vs. 5.5 months), although these results did not reach statistical significance (52). Similar findings regarding the relationship between bevacizumab-associated hypertension and improved survival have been reported in the CALGB 90206 trial involving patients with metastatic renal cell carcinoma (53). However, further investigation of this association is warranted. A recent presentation of a large study of approximately 5,900 patients across six placebo-controlled, phase III studies of bevacizumab showed hypertension arising during treatment did not predict improvement in PFS or OS (54).

A retrospective evaluation of risk factors associated with severe pulmonary hemorrhage in patients treated with carboplatin/paclitaxel plus bevacizumab suggests that baseline tumor cavitation was the only risk factor for early-onset pulmonary hemorrhage. Central tumor location was not predictive of risk (55).

## SMALL-MOLECULE, ANTIANGIOGENIC TYROSINE KINASE INHIBITORS (TKIs)

The proangiogenic activity of VEGF is dependent on signaling through its cognate receptors (i.e., the VEGFRs); thus, blocking these receptors is another antiangiogenic strategy (56). The receptors can be inhibited using small-molecule TKIs, which compete with adenosine triphosphate (ATP) for the active site of the tyrosine kinase (TK) domain and block receptor activation (56). Many TKIs that inhibit VEGFR also inhibit other key pathways involved in angiogenesis, including FGF and PDGF and their respective receptors (56). It has been suggested that because of some redundancy in proangiogenic signaling, both the FGF and PDGF pathways may play a role in the development of resistance to VEGF blockade (57-60). Thus, by targeting multiple pathways, these agents may have the potential to overcome resistance to agents directed against only VEGF, such as bevacizumab (61, 62).

### Sorafenib

Sorafenib is an oral multi-kinase inhibitor that targets tumor growth, survival, and angiogenesis, by inhibiting VEGFR2, VEGFR3, and PDGF receptor (PDGFR) TKs (63). It also targets the Raf kinases, key signaling molecules downstream of Ras that transmit proliferative and cell survival signals (63). Single-agent sorafenib has shown activity in patients with advanced NSCLC in the first-line setting (64, 68).

Sorafenib has been combined with conventional chemotherapy in multiple studies (Table 3) (66, 67). In one phase I/II trial, carboplatin/paclitaxel in combination with sorafenib was evaluated in patients with advanced NSCLC (66). Among 39 evaluable patients, nine achieved a PR, and one achieved a CR; however, all these patients had melanoma. Median PFS for patients without melanoma was 104 days. The drug-related AEs were similar to those reported with single-agent sorafenib and included rash, hand-foot syndrome, and GI side effects (66, 74). Based on these results, the randomized phase III ESCAPE trial was initiated. The ESCAPE trial enrolled 926 patients with advanced NSCLC who received carboplatin/paclitaxel with or without sorafenib as first-line therapy (67). There were no significant differences between the treatment arms in RR (24% vs. 27%), PFS (4.6 months vs. 5.4 months), or OS (10.7 months vs. 10.6 months), and as a result the trial was stopped early. There was a higher rate of drug-related infection in patients who received sorafenib than in those who received placebo (6.5% vs. 2.2%; *p =* .002). The grade 5 toxicity was observed more frequently in patients who received sorafenib versus those who received chemotherapy alone (14 patients vs. 4 patients;*p* < .001). In a subset analysis, shorter survival times were observed in patients with squamous cell histology who received sorafenib plus chemotherapy compared with those who received chemotherapy alone, though this observation was not statistically significant (67). Another ongoing large phase III trial, NExUS (NCT00449033), is evaluating gemcitabine/cisplatin with or without sorafenib as first-line therapy in patients with NSCLC (Table 3), and other studies are assessing second-line sorafenib mono therapy (Table 4).

**Table 3:** Results from phase II and III trials of antiangiogenic TKIs in combination with chemotherapy in NSCLC

Reference	Phase	Regimen	N^a^	RR (%)	PFS (mo)	OS (mo)
*Sorafenib*						
Flaherty 2008 (66)	I	CBDCA AUC 6 + PTX 225 mg/m^2^ + sorafenib 100, 200, or 400 mg twice daily.	39	26	10.1 (melanoma)	NR
					3.4 (other tumor types)	
Scagliotti 2010 (67)	III	CBDCA AUC 6 + PTX 225 mg/m^2^ + placebo.	462	24	5.4	10.6
		CBDCA AUC 6 + PTX 225 mg/m^2^ + sorafenib 400 mg twice daily.	464	27.4	4.6	10.7
*Cediranib*						
Gadgeel 2009 (68)	II	Pem 500 mg/m^2^ + cediranib 30 mg/day.	31	16	NR	NR
*Vandetanib*						
De Boer 2009 (69)	I	Pem 500 mg/m^2^ + vandetanib 100 mg/day.	10	10	NR	NR
		Pem 500 mg/m^2^ + vandetanib 300 mg/day.	11	0	NR	NR
Heymach 2008 (70)	II	CBDCA AUC 6 + PTX 200 mg/m^2^ + placebo.	52	25	∼6 (23 weeks)	12.6
		CBDCA AUC 6 + PTX 200 mg/m^2^ + vandetanib 300 mg/day.	56	32	∼6 (24 weeks)	10.2
Heymach 2007 (71)	II	TXT 75 mg/m^2^ + placebo.	41	12	2.8	13.4
		TXT 75 mg/m^2^ + vandetanib 100 mg/day.	42	26	4.3	13.1
		TXT 75 mg/m^2^ + vandetanib 300 mg/day.	44	18	4.0	7.9
Herbst 2010 (72)	III	TXT 75 mg/m^2^ + placebo.	697	10	3.2	9.9
		TXT 75 mg/m^2^ + vandetanib 100 mg/day.	694	17	4.0	10.3
De Boer 2009 (73)	III	Pem 500 mg/m^2^ + placebo.	278	7.9	NR	NR
		Pem 500 mg/m^2^ + vandetanib 100 mg/day.	256	19.1	NR	NR

a Patients evaluable for efficacy.

*Abbreviations*. AUC: area under the curve; CBDCA: carboplatin; NR: not reported; NSCLC: non-small cell lung cancer; OS: overall survival; Pem: pemetrexed; PFS: progressionfree survival; PTX: paclitaxel; RR: response rate; TKI: tyrosine kinase inhibitor; TXT: docetaxel.

**Table 4:** Overview of ongoing (actively recruiting) phase II and III trials of antiangiogenic TKIs in NSCLC^a^

Agent	Trial Description	Identifier Number
*Sorafenib*		
	Phase II trial of sorafenib in patients with advanced NSCLC after failure of EGFR-TKI.	NCT00922584
	Phase II trial of sorafenib in combination with erlotinib in patients with advanced NSCLC after failure of chemotherapy.	NCT00801385
	Phase II trial of sorafenib in patients with relapsed or refractory advanced NSCLC (nonsmokers or former light smokers).	NCT00754923
	Phase III trial of third- or fourth-line sorafenib versus placebo in patients with predominantly nonsquamous NSCLC.	NCT00863746
*Sunitinib*		
	Phase II trial of first-line sunitinib in patients aged >70 years with advanced NSCLC.	NCT00864721
	Phase II trial of second-line sunitinib and/or pemetrexed in patients with advanced NSCLC.	NCT00698815
	Phase II trial of sunitinib in combination with cisplatin and docetaxel as salvage therapy in patients with advanced NSCLC.	NCT01019798
	Phase II trial of sunitinib as maintenance therapy in patients with advanced NSCLC previously treated with combination chemotherapy.	NCT01210053
	Phase III trial of sunitinib versus placebo as maintenance therapy in patients with advanced NSCLC previously treated with combination chemotherapy.	NCT00693992
*Cediranib*		
	Phase III trial of first-line cediranib in combination with carboplatin and paclitaxel in patients with advanced NSCLC (BR. 29).	NCT00795340
	Phase II trial of second- or third-line cediranib in combination with pemetrexed in patients with advanced NSCLC.	NCT00410904
*Vandetanib*		
	Phase II trial of first-line vandetanib in combination with carboplatin and paclitaxel in patients with advanced NSCLC.	NCT00093392.
*BIBF 1120*		
	Phase III trial of second-line BIBF 1120 in combination with docetaxel versus docetaxel alone in patients with advanced NSCLC (LUME-Lung 1).	NCT00805194
	Phase III trial of second-line BIBF 1120 in combination with pemetrexed versus pemetrexed alone in patients with advanced nonsquamous NSCLC (LUME-Lung 2).	NCT00806819
*Pazopanib*		
	Phase II/III trial of adjuvant pazopanib versus placebo in patients with stage I surgically resected NSCLC.	NCT00775307
	Phase II trial of first-line pazopanib in combination with paclitaxel versus carboplatin in combination with paclitaxel in patients with advanced NSCLC.	NCT00866528
	Phase II trial of second-line pazopanib in combination with erlotinib versus erlotinib alone in patients with advanced NSCLC.	NCT01027598
	Phase II trial of third-line pazopanib in patients with advanced NSCLC.	NCT01049776
*Axitinib*		
	Phase II trial of first-line axitinib in combination with cisplatin and gemcitabine in patients with squamous NSCLC.	NCT00735904

a ClinicalTrials.gov accessed on November 22, 2010.

Abbreviations. EGFR: epidermal growth factor receptor; NSCLC: non-small cell lung cancer; TKI: tyrosine kinase inhibitor.

### Sunitinib

Sunitinib is a multitargeted small-molecule TKI that targets VEGFR1, VEGFR2, VEGFR3, PDGFR, fms-like TK-3 (Flt3), c-kit, and rearranged during transfection (RET) (75, 76). Sunitinib has shown single-agent activity in a multi-center phase II trial as second- or third-line therapy in patients with advanced NSCLC, when administered according to a schedule of 4 weeks with treatment followed by 2 weeks without treatment, at a starting dose of 50 mg/day (76). The trial resulted in a RR of 11%, PFS of 12 weeks, and OS of 23.4 weeks. These findings are comparable with those of currently approved agents in this treatment setting. The most common grade 3/4 nonhematologic AEs included fatigue/asthenia (29%), pain/myalgia (17%), dyspnea (11%), and nausea/vomiting (10%). The grade 3/4 hematologic AEs included lymphopenia (25%), thrombocytopenia (5%), and neutropenia (5%) (76). Notably, of the three patients in the study who suffered hemorrhage-related deaths, two had squamous NSCLC (both experienced pulmonary hemorrhage). In a separate, open-label phase II study, sunitinib was administered continuously (without a 2-week break) at a lower starting dose of 37.5 mg/day to 47 patients with advanced NSCLC as second- or third-line treatment *(77).* One patient achieved a PR, and 11 patients demonstrated SD. Median PFS and OS were 2.7 months and 8.6 months, respectively. The most frequently reported grade 3/4 AEs included fatigue (17.0%), hypertension (8.5%), and dyspnea (6.4%).

In a phase I study, sunitinib was combined with cisplatin and gemcitabine as first-line therapy in patients with advanced NSCLC (78). The combination resulted in a manageable toxicity profile and PRs were observed in five of 24 patients. A second phase I study evaluated the combination of sunitinib and docetaxel in 50 patients with advanced solid tumors, including 18 patients with NSCLC (79). PR was observed in three patients, SD was observed in 12 patients, and AEs were manageable. Ongoing clinical trials are further evaluating the benefits of adding sunitinib to standard therapies and as maintenance after first-line therapy (Table 4).

### Cediranib

Cediranib is a highly potent and selective inhibitor of the VEGF pathway with activity against all three VEGFRs, PDGFRs, and c-kit (80). In phase I studies, cediranib has demonstrated antitumor activity as a single agent with a manageable toxicity profile. The most frequently reported AEs include diarrhea, fatigue, dysphonia, and hypertension (81,82). Cediranib at doses of 20, 30, and 45 mg was evaluated in a phase I study in combination with four chemotherapy regimens (FOLFOX, irinotecan, docetaxel, and pemetrexed) in 46 heavily pretreated patients with advanced solid tumors (83). Of 35 patients who were evaluable for toxicity, grade 3/4 AEs observed across all four arms included fatigue, diarrhea, hand-foot syndrome, neutropenic fever, and hypertension (83). In another phase I study (N = 15), cediranib 30 mg and 45 mg was combined with standard doses of cis-platin/gemcitabine in patients with advanced NSCLC (84). The combination was associated with increased toxicity compared with chemotherapy alone (84). All 12 of the evaluable patients showed some degree of initial tumor shrinkage and four achieved PRs (84). In another phase I trial (N = 20), once-daily cediranib 30 mg and 45 mg in combination with carboplatin/paclitaxel administered every 3 weeks was evaluated as first-line treatment in patients with advanced NSCLC (85). Patients receiving anticoagulation were eligible, but patients with a history of hemoptysis or bleeding were excluded. Adverse events included fatigue, myalgia, hypertension, GI toxicities, and neutropenia (85). Progression-free survival was reported in nine patients, and all but one patient showed some evidence of tumor shrinkage; median TTP was 7.6 months (85). Antitumor activity was observed at both dose levels, but with no indication of a dose effect (85).

BR .24 is a randomized, double-blind, placebo-controlled phase II/III trial of cediranib 30 mg in combination with carboplatin/paclitaxel as first-line treatment in patients with NSCLC (86). This trial was not continued into phase III as there appeared to be excessive toxicity, although evidence of clinical activity was observed (86, 87). BR.29 will compare cediranib 20 mg in combination with carboplatin/paclitaxel with chemotherapy alone as first-line treatment in patients with NSCLC (Table 4).

An ongoing phase II trial is evaluating cediranib in combination with pemetrexed for second- or third-line treatment of advanced NSCLC (Table 3) (68). This study consists of two cohorts: patients who have not received prior beva-cizumab (cohort A) and patients who have received prior be-vacizumab (cohort B). In a preliminary analysis of the first 31 evaluable patients, the confirmed RR was 16% (10% in cohort A and 25% in cohort B) and the disease control rate was 71% (74% in cohort A and 67% in cohort B). The grade 3/4 nonhematologic AEs included fatigue (21%), diarrhea (9%), anorexia (6%), hypertension (3%), cardiac ischemia (3%), bronchopleural fistula (3%), and esophagitis (3%). The grade 3/4 neutropenia was reported in 21% of patients and febrile neutropenia occurred in 3% of patients (68).

### Vandetanib

Vandetanib is a small-molecule inhibitor that blocks both the VEGFR and EGFR pathways, although it is more specific for the VEGFR pathway (88, 89). It is also a potent inhibitor of RET receptor TK activity (72). A number of studies have evaluated vandetanib in combination with chemotherapy (Table 3) (69-73). Response rates were increased in the combination arms compared to chemotherapy-only arms. In addition, most of these studies demonstrated a prolongation in PFS, but no improvement in OS. In a double-blind randomized, phase 3 trial (ZODIAC) the combination of vandetanib and docetaxel was evaluated as a second-line treatment in patients with advanced NSCLC (N = 1,391). The addition of vandetanib improved PFS (4.0 months in the Vandetanib group vs. 3.2 months in the placebo group) but no significant improvement in OS was reported (72). ZEIST, a randomized phase III trial (N = 1,240) in patients with advanced, previously treated NSCLC, demonstrated that single-agent vandetanib and erlotinib had equivalent efficacy by PFS (HR, 0.98; *p* = .721) and OS (HR, 1.01; p = .830), but that vandetanib was associated with a higher incidence of toxicity (90). Vandetanib is also being evaluated in the phase III trial (ZEPHYR) in patients with advanced NSCLC who have progressed after treatment with chemotherapy and an EGFR TKI, but preliminary results indicate that the trial did not reach its primary end point of OS (91). Regulatory submissions for vandetanib in patients with NSCLC have also been withdrawn (91).

### BIBF1120

BIBF 1120 is a TKI targeting VEGFR1, VEGFR2, VEGFR3, FGFR1, FGFR2, FGFR3, and PDGFR, with the potential to inhibit proangiogenic signaling pathways in vascular en-dothelial cells, pericytes, and smooth-muscle cells (92). BIBF 1120 was evaluated as a single agent in a phase II trial in 73 patients with advanced NSCLC who had received one to two prior chemotherapeutic regimens (93). Patients were randomly assigned to receive twice-daily BIBF 1120, 150 mg (N = 37) or 250 mg (N = 36). Median PFS was 1.6 months in the overall population (N = 73) and 2.9 months in patients with an ECOG performance status (PS) 0 to 1 (N = 57), while median OS was 22 weeks in the overall population and 38 weeks in patients with PS 0 to 1. One PR was achieved, and the SD rate was 48% for all patients and 59% in patients with a PS of 0 to 1. The grade 3/4 toxicities included reversible alanine aminotransferase (ALT) elevations (9.6%), diarrhea (9.6%), nausea (8.2%), fatigue (5.5%), and vomiting (4.1%) (93). BIBF 1120 was not associated with a high frequency of hypertension, which is commonly reported with other VEGF inhibitors (46, 93).

Results from phase I studies confirmed the feasibility of combining BIBF 1120 with chemotherapy (94, 95). In a phase I trial (N = 25), BIBF 1120 in combination with carboplatin/paclitaxel was evaluated in chemo-naive patients with advanced NSCLC (94). The maximum tolerated dose (MTD) of BIBF 1120 was 200 mg twice daily, and no clinically relevant changes to carboplatin/paclitaxel pharmacoki-netic parameters were observed (94). In another phase I dose-escalation study, BIBF 1120 plus pemetrexed (500 mg/m^2^) was administered to 26 patients with NSCLC who had received prior first-line, platinum-based chemotherapy (95). The MTD of BIBF 1120, in combination with standard-dose pemetrexed, was 200 mg twice daily, with no clinically relevant effects of BIBF 1120 pharmacokinetics observed in combination with pemetrexed (95). During the first treatment cycle, dose-limiting toxicities (DLTs) (all grade 3) occurred in seven patients for all doses and included transaminase elevations, fatigue, confusion, anorexia, and GI disorders (95). Among the 26 evaluable patients, one patient achieved a CR and 13 (50%) patients had SD. Based on these data, two randomized phase III studies are under way to evaluate BIBF 1120 in combination with docetaxel or pemetrexed for patients with advanced NSCLC after failure of first-line therapy (Table 4).

### Pazopanib

Pazopanib is a multitargeted TKI that blocks VEGFR1, VEGFR2, VEGFR3, PDGFR, and c-kit (96). Preclinical studies indicate that pazopanib is effective in inhibiting angiogen-esis (97). Pazopanib has been used as neoadjuvant monother-apy in patients with early-stage NSCLC (98). Of a total of 35 patients, three achieved a PR. The grade 3 toxicities were observed in five patients and included ALT elevations, hypertension, dyspnea, pneumonia, urinary tract infection, rash, increase in blood potassium, and lymphopenia. One patient experienced grade 4 bilateral pulmonary emboli 11 days after surgery (98). In a separate exploratory analysis of cytokines and angiogenic factors (C/AFs) in the serum of patients with early-stage NSCLC who received preoperative treatment with pazopanib, significant changes in eight C/AFs were reported (99). In particular, plasma levels of VEGFR2 (*p* < .0001) and placental growth factor (PIGF;*p* < .0001) were significantly decreased after pazopanib treatment. There was also a correlation between serum levels of VEGFR2 and tumor shrinkage (*p* < .05), suggesting its potential for use as a predictive marker of response (99). The efficacy and tolerability of pazopanib in advanced NSCLC, either alone or in combination with chemotherapy, is being evaluated in numerous clinical trials (Table 4).

### Axitinib

Axitinib is a potent small-molecule TKI of VEGFR1, VEGFR2, VEGFR3, PDGR, and c-kit (100). In a phase I trial in 36 patients with advanced solid tumors, single-agent axitinib demonstrated antitumor activity in multiple tumor types, including NSCLC (101). The most common toxicities of any grade were hypertension (61%), fatigue (28%), nausea (19%), and diarrhea (17%) (101). Based on these data, a phase II study was conducted in 32 patients with advanced NSCLC (102). Nine patients had not received prior chemotherapy for metastatic disease and 23 patients received > 1 prior regimen (102). Response rate was 9% and median PFS was 4.9 months in the overall study population and 9.2 months in treatment-naive patients. Median OS was 14.8 months in the overall population and 14.8 months in patients who received axitinib as first-line therapy. One-year survival rates were 57% and 78%, respectively. Of the grade 1 or 2 AEs that occurred in at least 15% of patients, those that also occurred at grade 3 severity included fatigue, hypertension, diarrhea, and vomiting (102).

Axitinib was combined with standard paclitaxel/ carboplatin and gemcitabine/cisplatin chemotherapy in a phase I trial of 47 patients with NSCLC and other solid tumors (103). Response rate in the paclitaxel/carboplatin cohort was 29%, and in the gemcitabine/cisplatin cohort RR was 26% (103). The dose-limiting toxicities included fatigue, proteinuria, and rashes (103). A subcohort analysis of patients with squamous cell histology showed that axitinib plus paclitaxel/carboplatin was well tolerated, with no evidence of grade >3 hemoptysis (103). Trials with single-agent axitinib and combination therapy with axitinib in advanced NSCLC are ongoing (Tables 3 and 4).

## CONCLUSIONS

The availability of treatment options for patients with advanced NSCLC has expanded. As seen in the E4599 trial of bevacizumab, therapy aimed at blocking angiogenesis can be effective when combined with standard doublet chemotherapy in patients with NSCLC. While a large number of patients were initially excluded from bevacizumab treatment because of safety concerns, some of these patients are now eligible, including patients with brain metastases and those receiving anticoagulation therapy. Patients with squamous histology remain ineligible. Subgroup analyses have shown limited efficacy and increased toxicity in the elderly, in whom bevacizumab should be used with caution.

Several multitargeted, antiangiogenic TKIs in clinical development for NSCLC have shown feasibility for combination with standard chemotherapy. The clinical advantages of this class of drugs are their oral administration and activity against multiple targets. However, improved OS in combination with chemotherapy has yet to be demonstrated in phase III trials. To date, toxicity profiles with these agents seem acceptable. Large phase III trials will determine the role of these agents in the treatment of patients with advanced NSCLC.

How do we personalize treatment strategies with antiangiogenic agents to achieve maximal efficacy with minimal toxicity? The identification of effective biomarkers will be critical. Several biomarkers have already been evaluated (although not validated) and may be predictive of treatment benefit. The development of hypertension may also be a surrogate marker of efficacy in patients treated with bevacizumab. Molecular markers and genetic mapping will be important for individualizing treatment regimens, predicting which patients might benefit from specific regimens, and evaluating the efficacy of specific antiangiogenic therapies.

*Prior publication statement:* This manuscript has neither been published nor submitted for publication elsewhere.
